# Tetra­ethyl­ammonium tricarbonyl­chlorido­(pyrazine-2-carboxyl­ato-*N* ^1^,*O*)rhenate(I)

**DOI:** 10.1107/S1600536809042160

**Published:** 2009-10-17

**Authors:** Janine Suthiram, Kanyisa Mhlaba, Jan Rijn Zeevaart, Hendrik G. Visser, Andreas Roodt

**Affiliations:** a Radiochemistry, South African Nuclear Energy Corporation Ltd. (Necsa), PO Box 582, Pretoria, 0001, South Africa; b Department of Chemistry, University of the Free State, PO Box 339, Bloemfontein, 9300, South Africa

## Abstract

In the title complex, (C_8_H_20_N)[Re(C_5_H_3_N_2_O_2_)Cl(CO)_3_], the Re^I^ atom is coordinated facially by three carbonyl groups; the bidentate pyrazine­carboxyl­ato ligand and a chlorine atom complete the distorted octa­hedral coordination.

## Related literature

For synthetic background, see: Alberto *et al.* (1996 ▶). For related structures, see: Schutte *et al.* (2008 ▶); Kemp (2006 ▶); Wang *et al.* (2003 ▶); Alvarez *et al.* (2007 ▶); Brasey *et al.* (2004 ▶); Mundwiler *et al.* (2004 ▶). For bond-length data, see: Allen *et al.* (1987 ▶).
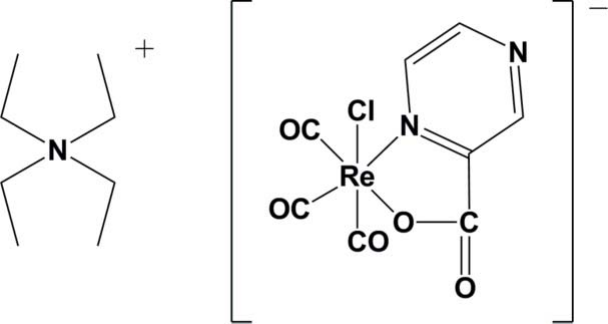


## Experimental

### 

#### Crystal data

(C_8_H_20_N)[Re(C_5_H_3_N_2_O_2_)Cl(CO)_3_]
*M* *_r_* = 559.02
Monoclinic, 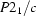

*a* = 7.927 (5) Å
*b* = 22.278 (5) Å
*c* = 10.903 (5) Å
β = 90.506 (5)°
*V* = 1925.4 (16) Å^3^
*Z* = 4
Mo *K*α radiation
μ = 6.48 mm^−1^
*T* = 100 K
0.27 × 0.20 × 0.11 mm

#### Data collection

Bruker X8 APEXII 4K Kappa CCD diffractometer
Absorption correction: multi-scan (*SADABS*; Bruker, 2004 ▶) *T*_min_ = 0.273, *T*_max_ = 0.539
32482 measured reflections
4781 independent reflections
4121 reflections with *I* > 2σ(*I*)
*R*_int_ = 0.046

#### Refinement

*R*[*F* ^2^ > 2σ(*F* ^2^)] = 0.021
*wR*(*F* ^2^) = 0.098
*S* = 1.18
4781 reflections
235 parameters
H-atom parameters constrained
Δρ_max_ = 1.05 e Å^−3^
Δρ_min_ = −1.38 e Å^−3^

### 

Data collection: *APEX2* (Bruker, 2005 ▶); cell refinement: *SAINT-Plus* (Bruker, 2004 ▶); data reduction: *SAINT-Plus* and *XPREP* (Bruker, 2004 ▶); program(s) used to solve structure: *SHELXS97* (Sheldrick, 2008 ▶); program(s) used to refine structure: *SHELXL97* (Sheldrick, 2008 ▶); molecular graphics: *DIAMOND* (Brandenburg & Putz, 2005 ▶); software used to prepare material for publication: *WinGX* (Farrugia, 1999 ▶).

## Supplementary Material

Crystal structure: contains datablocks global, I. DOI: 10.1107/S1600536809042160/pv2218sup1.cif

Structure factors: contains datablocks I. DOI: 10.1107/S1600536809042160/pv2218Isup2.hkl

Additional supplementary materials:  crystallographic information; 3D view; checkCIF report

## Figures and Tables

**Fig. 1.**
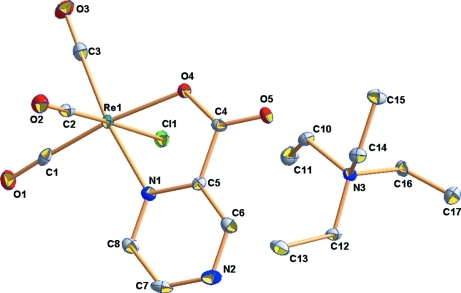
A view of the title complex plotted with 50% probability displacement ellipsoids; hydrogen atoms have been omitted for clarity.

## supplementary crystallographic information

### Comment

The title complex, (I), forms a part of an ongoing investigation of the structural and kinetic behaviour of *fac*-Re(CO)_3_ compounds (Schutte *et al.*, 2008). It crystallized as an anionic Re^I^ compound and one tetraethylammonium counter ion in the asymmetric unit (Fig. 1). The Re—CO bond distances are well within the normal range (Allen *et al.*, 1987). The small bite angle O4—Re1—N1 might be a reason for the slightly distorted octahedral geometry around the metal centre. There are no classical hydrogen bonds in the structure.

### Experimental

ReCl_3_(CO)_3_ (64.2 mg, 0.01 mmol) was suspended in 10 ml methanol. The solution was heated to reflux and 2-pyrazinecarboxylic acid (13.1 mg, 0.01 mmol) dissolved in *ca* 5 ml methanol was added whilst stirring. A bright yellow colour resulted on addition of the ligand to the metal. K_2_CO_3_ 7.1 mg (0.005 mmol) was added to the solution. The reaction solution was refluxed for 6 h after which the solvent volume was decreased on a rotovapor. The MeOH solution was layered with a minimal amount of diethyl ether and left to stand in a refrigerator. After a few days yellow crystals were formed.

### Refinement

The methyl, methylene and aromatic H atoms were placed in geometrically idealized positions with C—H distances = 0.96, 0.97 and 0.96 Å, respectively, and constrained to ride on their parent atoms, with *U*_iso_(H) = 1.5*U*_eq_(methyl-C) and 1.2*U*_eq_(methylene and aromatic-C). The highest residual electron density was located 0.93 Å from H17C and was essentially meaningless.

### Crystal data

**Table:**

(C_8_H_20_N)[Re(C_5_H_3_N_2_O_2_)Cl(CO)_3_]	*F*(000) = 1088
*M**_r_* = 559.02	*D*_x_ = 1.929 Mg m^−^^3^
Monoclinic, *P*2_1_/*c*	Mo *K*α radiation, λ = 0.71073 Å
Hall symbol: -P 2ybc	Cell parameters from 9901 reflections
*a* = 7.927 (5) Å	θ = 2.7–28.3°
*b* = 22.278 (5) Å	µ = 6.48 mm^−^^1^
*c* = 10.903 (5) Å	*T* = 100 K
β = 90.506 (5)°	Cuboid, yellow
*V* = 1925.4 (16) Å^3^	0.27 × 0.20 × 0.11 mm
*Z* = 4	

### Data collection

**Table:**

Bruker X8 APEXII 4K Kappa CCD diffractometer	4781 independent reflections
Radiation source: sealed tube	4121 reflections with *I* > 2σ(*I*)
graphite	*R*_int_ = 0.046
phi and ω scans	θ_max_ = 28.3°, θ_min_ = 1.8°
Absorption correction: multi-scan (*SADABS*; Bruker, 2004)	*h* = −10→9
*T*_min_ = 0.273, *T*_max_ = 0.539	*k* = −29→29
32482 measured reflections	*l* = −14→13

### Refinement

**Table:**

Refinement on *F*^2^	0 restraints
Least-squares matrix: full	H-atom parameters constrained
*R*[*F*^2^ > 2σ(*F*^2^)] = 0.021	*w* = 1/[σ^2^(*F*_o_^2^) + (0.0591*P*)^2^] where *P* = (*F*_o_^2^ + 2*F*_c_^2^)/3
*wR*(*F*^2^) = 0.098	(Δ/σ)_max_ = 0.001
*S* = 1.18	Δρ_max_ = 1.05 e Å^−^^3^
4781 reflections	Δρ_min_ = −1.38 e Å^−^^3^
235 parameters	

### Special details

**Table:**

Geometry. All e.s.d.'s (except the e.s.d. in the dihedral angle between two l.s. planes) are estimated using the full covariance matrix. The cell e.s.d.'s are taken into account individually in the estimation of e.s.d.'s in distances, angles and torsion angles; correlations between e.s.d.'s in cell parameters are only used when they are defined by crystal symmetry. An approximate (isotropic) treatment of cell e.s.d.'s is used for estimating e.s.d.'s involving l.s. planes.

### Fractional atomic coordinates and isotropic or equivalent isotropic displacement parameters (Å^2^)

**Table:**

	*x*	*y*	*z*	*U*_iso_*/*U*_eq_	
Re1	0.30393 (2)	0.198197 (7)	0.407485 (15)	0.01064 (8)	
Cl1	0.29768 (13)	0.09512 (5)	0.49378 (10)	0.0154 (2)	
C5	0.3651 (5)	0.13259 (19)	0.1726 (4)	0.0117 (8)	
O5	0.0904 (4)	0.10473 (15)	0.1121 (3)	0.0186 (7)	
N3	0.1752 (4)	−0.07955 (16)	0.2734 (3)	0.0111 (7)	
O4	0.1219 (4)	0.16145 (14)	0.2811 (3)	0.0143 (7)	
N2	0.6136 (5)	0.10700 (19)	0.0608 (4)	0.0198 (9)	
N1	0.4559 (5)	0.15900 (16)	0.2633 (3)	0.0132 (8)	
C14	0.1057 (5)	−0.0443 (2)	0.1656 (4)	0.0146 (9)	
H14B	0.1568	−0.0047	0.1666	0.018*	
H14A	0.1403	−0.064	0.0906	0.018*	
C4	0.1762 (5)	0.1323 (2)	0.1880 (4)	0.0131 (9)	
C10	0.1294 (6)	−0.0471 (2)	0.3912 (4)	0.0169 (9)	
H10A	0.1667	−0.0058	0.3849	0.02*	
H10B	0.0075	−0.0467	0.3983	0.02*	
C15	−0.0836 (6)	−0.0368 (2)	0.1619 (4)	0.0182 (10)	
H15B	−0.1153	−0.0138	0.0909	0.027*	
H15C	−0.1197	−0.0164	0.2345	0.027*	
H15A	−0.1361	−0.0756	0.1578	0.027*	
C12	0.3647 (5)	−0.0855 (2)	0.2602 (4)	0.0147 (9)	
H12B	0.4069	−0.1106	0.3264	0.018*	
H12A	0.3875	−0.1062	0.1838	0.018*	
C17	0.1141 (6)	−0.1786 (2)	0.1620 (5)	0.0215 (10)	
H17B	0.0637	−0.2173	0.1739	0.032*	
H17A	0.231	−0.1835	0.1418	0.032*	
H17C	0.0569	−0.1581	0.0962	0.032*	
C1	0.4817 (6)	0.2209 (2)	0.5162 (4)	0.0160 (9)	
C7	0.7019 (6)	0.1324 (2)	0.1527 (4)	0.0187 (10)	
H7	0.819	0.1323	0.149	0.022*	
C16	0.0997 (6)	−0.14213 (19)	0.2785 (4)	0.0139 (9)	
H16A	0.1545	−0.1641	0.3445	0.017*	
H16B	−0.0188	−0.1386	0.2988	0.017*	
C13	0.4627 (6)	−0.0272 (2)	0.2612 (5)	0.0228 (11)	
H13C	0.5808	−0.0355	0.2525	0.034*	
H13B	0.4444	−0.0067	0.3374	0.034*	
H13A	0.425	−0.0023	0.1945	0.034*	
C8	0.6250 (6)	0.1586 (2)	0.2526 (4)	0.0167 (9)	
H8	0.6913	0.1764	0.3134	0.02*	
C6	0.4450 (6)	0.1077 (2)	0.0729 (4)	0.0169 (9)	
H6	0.3791	0.0906	0.0111	0.02*	
C11	0.2032 (6)	−0.0737 (2)	0.5073 (4)	0.0227 (11)	
H11B	0.1677	−0.0504	0.5765	0.034*	
H11A	0.3241	−0.0733	0.5028	0.034*	
H11C	0.1646	−0.1143	0.5162	0.034*	
C3	0.1409 (6)	0.2291 (2)	0.5218 (4)	0.0148 (9)	
O2	0.3224 (4)	0.32482 (15)	0.2989 (3)	0.0206 (7)	
O3	0.0411 (4)	0.24882 (16)	0.5855 (3)	0.0215 (7)	
O1	0.5931 (4)	0.23294 (16)	0.5808 (3)	0.0241 (8)	
C2	0.3140 (5)	0.2765 (2)	0.3371 (4)	0.0139 (9)	

### Atomic displacement parameters (Å^2^)

**Table:**

	*U*^11^	*U*^22^	*U*^33^	*U*^12^	*U*^13^	*U*^23^
Re1	0.01130 (11)	0.00960 (12)	0.01101 (12)	−0.00026 (6)	−0.00005 (7)	−0.00013 (6)
Cl1	0.0170 (5)	0.0123 (5)	0.0169 (5)	−0.0006 (4)	−0.0002 (4)	0.0005 (4)
C5	0.013 (2)	0.010 (2)	0.012 (2)	0.0021 (16)	−0.0005 (16)	0.0015 (16)
O5	0.0130 (16)	0.0202 (19)	0.0227 (17)	−0.0029 (13)	−0.0034 (13)	−0.0071 (14)
N3	0.0113 (17)	0.0110 (18)	0.0110 (17)	−0.0010 (14)	0.0013 (13)	0.0012 (14)
O4	0.0127 (15)	0.0161 (17)	0.0141 (15)	−0.0004 (12)	−0.0006 (12)	−0.0029 (13)
N2	0.017 (2)	0.026 (2)	0.0168 (19)	0.0039 (17)	0.0031 (16)	0.0009 (17)
N1	0.0121 (17)	0.0103 (19)	0.0172 (19)	−0.0001 (14)	0.0000 (14)	0.0036 (15)
C14	0.015 (2)	0.013 (2)	0.015 (2)	0.0012 (17)	0.0014 (17)	0.0052 (17)
C4	0.011 (2)	0.013 (2)	0.015 (2)	0.0013 (16)	0.0001 (16)	0.0019 (17)
C10	0.014 (2)	0.017 (2)	0.019 (2)	0.0022 (18)	0.0026 (18)	−0.0043 (19)
C15	0.015 (2)	0.024 (3)	0.016 (2)	0.0029 (19)	−0.0025 (17)	0.0024 (19)
C12	0.010 (2)	0.017 (2)	0.018 (2)	0.0019 (17)	0.0027 (16)	−0.0006 (18)
C17	0.023 (3)	0.020 (3)	0.022 (3)	0.000 (2)	0.002 (2)	−0.005 (2)
C1	0.023 (2)	0.009 (2)	0.016 (2)	0.0009 (18)	0.0019 (18)	−0.0015 (18)
C7	0.010 (2)	0.026 (3)	0.021 (2)	0.0035 (18)	0.0040 (17)	0.006 (2)
C16	0.014 (2)	0.010 (2)	0.017 (2)	−0.0034 (17)	0.0005 (17)	0.0017 (18)
C13	0.012 (2)	0.022 (3)	0.034 (3)	−0.0006 (19)	0.000 (2)	0.004 (2)
C8	0.015 (2)	0.018 (2)	0.017 (2)	0.0012 (18)	−0.0034 (17)	0.0026 (18)
C6	0.015 (2)	0.019 (3)	0.017 (2)	−0.0002 (18)	−0.0019 (17)	0.0016 (18)
C11	0.024 (3)	0.029 (3)	0.015 (2)	0.002 (2)	−0.0004 (19)	−0.005 (2)
C3	0.018 (2)	0.012 (2)	0.015 (2)	−0.0024 (17)	−0.0025 (18)	0.0039 (18)
O2	0.0225 (18)	0.0123 (17)	0.0272 (19)	−0.0016 (14)	0.0033 (15)	0.0027 (15)
O3	0.0279 (18)	0.0198 (19)	0.0170 (17)	0.0012 (15)	0.0096 (14)	−0.0011 (14)
O1	0.0222 (18)	0.021 (2)	0.0284 (19)	0.0010 (15)	−0.0097 (15)	−0.0060 (16)
C2	0.012 (2)	0.018 (2)	0.012 (2)	0.0026 (17)	0.0006 (16)	−0.0006 (18)

### Geometric parameters (Å, °)

**Table:**

Re1—C1	1.902 (5)	C15—H15C	0.96
Re1—C2	1.908 (5)	C15—H15A	0.96
Re1—C3	1.930 (5)	C12—C13	1.514 (6)
Re1—O4	2.149 (3)	C12—H12B	0.97
Re1—N1	2.172 (4)	C12—H12A	0.97
Re1—Cl1	2.4822 (12)	C17—C16	1.513 (6)
C5—N1	1.353 (6)	C17—H17B	0.96
C5—C6	1.379 (6)	C17—H17A	0.96
C5—C4	1.508 (6)	C17—H17C	0.96
O5—C4	1.231 (5)	C1—O1	1.156 (5)
N3—C14	1.514 (5)	C7—C8	1.383 (7)
N3—C12	1.517 (5)	C7—H7	0.93
N3—C16	1.518 (5)	C16—H16A	0.97
N3—C10	1.521 (5)	C16—H16B	0.97
O4—C4	1.282 (5)	C13—H13C	0.96
N2—C7	1.342 (6)	C13—H13B	0.96
N2—C6	1.344 (6)	C13—H13A	0.96
N1—C8	1.347 (6)	C8—H8	0.93
C14—C15	1.510 (6)	C6—H6	0.93
C14—H14B	0.97	C11—H11B	0.96
C14—H14A	0.97	C11—H11A	0.96
C10—C11	1.510 (6)	C11—H11C	0.96
C10—H10A	0.97	C3—O3	1.145 (5)
C10—H10B	0.97	O2—C2	1.156 (6)
C15—H15B	0.96		
			
C1—Re1—C2	88.49 (19)	H15B—C15—H15C	109.5
C1—Re1—C3	89.99 (19)	C14—C15—H15A	109.5
C2—Re1—C3	87.95 (19)	H15B—C15—H15A	109.5
C1—Re1—O4	172.22 (16)	H15C—C15—H15A	109.5
C2—Re1—O4	96.95 (16)	C13—C12—N3	115.6 (4)
C3—Re1—O4	95.74 (16)	C13—C12—H12B	108.4
C1—Re1—N1	98.34 (17)	N3—C12—H12B	108.4
C2—Re1—N1	92.94 (16)	C13—C12—H12A	108.4
C3—Re1—N1	171.64 (16)	N3—C12—H12A	108.4
O4—Re1—N1	75.91 (13)	H12B—C12—H12A	107.4
C1—Re1—Cl1	91.55 (14)	C16—C17—H17B	109.5
C2—Re1—Cl1	178.07 (14)	C16—C17—H17A	109.5
C3—Re1—Cl1	93.98 (13)	H17B—C17—H17A	109.5
O4—Re1—Cl1	82.82 (9)	C16—C17—H17C	109.5
N1—Re1—Cl1	85.15 (10)	H17B—C17—H17C	109.5
N1—C5—C6	120.4 (4)	H17A—C17—H17C	109.5
N1—C5—C4	116.3 (4)	O1—C1—Re1	177.5 (4)
C6—C5—C4	123.3 (4)	N2—C7—C8	122.4 (4)
C14—N3—C12	109.0 (3)	N2—C7—H7	118.8
C14—N3—C16	111.4 (3)	C8—C7—H7	118.8
C12—N3—C16	108.3 (3)	C17—C16—N3	115.4 (4)
C14—N3—C10	108.7 (3)	C17—C16—H16A	108.4
C12—N3—C10	111.5 (3)	N3—C16—H16A	108.4
C16—N3—C10	108.0 (3)	C17—C16—H16B	108.4
C4—O4—Re1	118.2 (3)	N3—C16—H16B	108.4
C7—N2—C6	115.8 (4)	H16A—C16—H16B	107.5
C8—N1—C5	117.2 (4)	C12—C13—H13C	109.5
C8—N1—Re1	128.6 (3)	C12—C13—H13B	109.5
C5—N1—Re1	114.1 (3)	H13C—C13—H13B	109.5
C15—C14—N3	115.6 (4)	C12—C13—H13A	109.5
C15—C14—H14B	108.4	H13C—C13—H13A	109.5
N3—C14—H14B	108.4	H13B—C13—H13A	109.5
C15—C14—H14A	108.4	N1—C8—C7	121.1 (4)
N3—C14—H14A	108.4	N1—C8—H8	119.4
H14B—C14—H14A	107.4	C7—C8—H8	119.4
O5—C4—O4	126.7 (4)	N2—C6—C5	123.1 (4)
O5—C4—C5	118.0 (4)	N2—C6—H6	118.5
O4—C4—C5	115.3 (4)	C5—C6—H6	118.5
C11—C10—N3	115.3 (4)	C10—C11—H11B	109.5
C11—C10—H10A	108.4	C10—C11—H11A	109.5
N3—C10—H10A	108.4	H11B—C11—H11A	109.5
C11—C10—H10B	108.4	C10—C11—H11C	109.5
N3—C10—H10B	108.4	H11B—C11—H11C	109.5
H10A—C10—H10B	107.5	H11A—C11—H11C	109.5
C14—C15—H15B	109.5	O3—C3—Re1	177.0 (4)
C14—C15—H15C	109.5	O2—C2—Re1	177.3 (4)
